# ImmunoGlobe: enabling systems immunology with a manually curated intercellular immune interaction network

**DOI:** 10.1186/s12859-020-03702-3

**Published:** 2020-08-10

**Authors:** Michelle B. Atallah, Varun Tandon, Kamir J. Hiam, Hunter Boyce, Michelle Hori, Waleed Atallah, Matthew H. Spitzer, Edgar Engleman, Parag Mallick

**Affiliations:** 1 Canary Center at Stanford, Department of Radiology, Stanford University School of Medicine, Stanford, CA USA; 2 Stanford University, Stanford, CA USA; 3 Departments of Otolaryngology and Microbiology & Immunology, Helen Diller Family Comprehensive Cancer Center, Parker Institute for Cancer Immunotherapy, Chan Zuckerberg Biohub, University of California, San Francisco, CA USA; 4 Department of Pathology, Stanford University School of Medicine, Stanford, CA USA

**Keywords:** Systems immunology, Immune network analysis, Cytokines, Immunobiology, Bioinformatics, Protein networks

## Abstract

**Background:**

While technological advances have made it possible to profile the immune system at high resolution, translating high-throughput data into knowledge of immune mechanisms has been challenged by the complexity of the interactions underlying immune processes. Tools to explore the immune network are critical for better understanding the multi-layered processes that underlie immune function and dysfunction, but require a standardized network map of immune interactions. To facilitate this we have developed ImmunoGlobe, a manually curated intercellular immune interaction network extracted from Janeway’s Immunobiology textbook.

**Results:**

ImmunoGlobe is the first graphical representation of the immune interactome, and is comprised of 253 immune system components and 1112 unique immune interactions with detailed functional and characteristic annotations. Analysis of this network shows that it recapitulates known features of the human immune system and can be used uncover novel multi-step immune pathways, examine species-specific differences in immune processes, and predict the response of immune cells to stimuli. ImmunoGlobe is publicly available through a user-friendly interface at www.immunoglobe.org and can be downloaded as a computable graph and network table.

**Conclusion:**

While the fields of proteomics and genomics have long benefited from network analysis tools, no such tool yet exists for immunology. ImmunoGlobe provides a ground truth immune interaction network upon which such tools can be built. These tools will allow us to predict the outcome of complex immune interactions, providing mechanistic insight that allows us to precisely modulate immune responses in health and disease.

## Introduction

The immune system is composed of a complex network of cells [1], receptors [2, 3] and secreted molecules [4], and an effective immune response requires coordination across these many components [3, 5, 6]. Consequently, the study of immune function and dysfunction at the level of pathways rather than individual components is critical in order to predict the outcome of immune interactions and precisely modulate immune responses. Knowledge of the underlying interaction network is therefore essential to the understanding of these immune responses, but its sheer complexity presents a barrier even to seasoned immunologists [7].

Recently, high-throughput technologies such as mass cytometry and gene expression profiling have enabled the measurement of immune responses in unprecedented detail [8, 9] by increasing the number of immune parameters that can be measured simultaneously. However, the lack of a foundational framework that integrates across the diverse components of the immune system has made it challenging to develop detailed, causal models explaining immune function and dysfunction [7, 10, 11]. Individual systems immunology approaches have been successful in several cases, for example in elucidating the immune networks involved in inflammation by using cytokine secretion profiles to create mathematical models of immune cell interactions [12] and inferring the immune networks that define tumor immunogenicity using genomic and transcriptomic data [13]. However, these types of systems immunology studies largely involve personalized analysis pipelines which require high levels of specialized analytical expertise to design and run, making them inaccessible to most researchers.

Growing recognition of the importance of such systems immunology approaches has resulted in the creation of a number of resources to address this issue, including analyses of the genes involved in various immune pathways [14], interactions between immune cells and cytokines [4], and proteomic analysis of known and potential cell:cell interactions [2]. While existing studies have contributed to our understanding of how parts of the immune system interact, there does not yet exist a comprehensive, gold-standard network map of the immune system that includes the variety of components that participate in immune responses, along with functional immune pathway annotations at the intercellular level. Such a map could act as the basis for the development of broadly applicable immune network analysis tools, such as those that exist for genomics and proteomics (for example DAVID [15] and KEGG [16]), which enable researchers to easily extract functional pathway-level information from high-throughput data.

Here we present ImmunoGlobe, available to the public at www.immunoglobe.org. ImmunoGlobe is a map of the immune intercellular interactome based on a widely-used and comprehensive immunology text [17, 18] that describes how components of the immune system interact to drive immune responses. By structuring our knowledge of immune interactions into a directional graph, ImmunoGlobe enables the easy querying of immune pathways and examination of the interactions between immune system components. By establishing a ground truth network of immune interactions, we anticipate that this resource will accelerate the development of immune network analysis tools, ultimately enabling the development of agents that can more precisely manipulate the immune response by accurately predicting the outcome of immune interactions.

## Results

### The ImmunoGlobe immune interaction network codifies immune interactions described in Janeway’s Immunobiology

To construct a comprehensive immune interaction network, we manually curated the 2799 immune interactions (edges) published in *Janeway’s Immunobiology* [18], widely regarded as an essential and comprehensive immunology text [17]. The data in this textbook is derived directly from the research literature, and focuses on physiologic functioning of the immune system rather than rare or atypical phenomena that may result from some experimental setups.

**Fig. 1:**
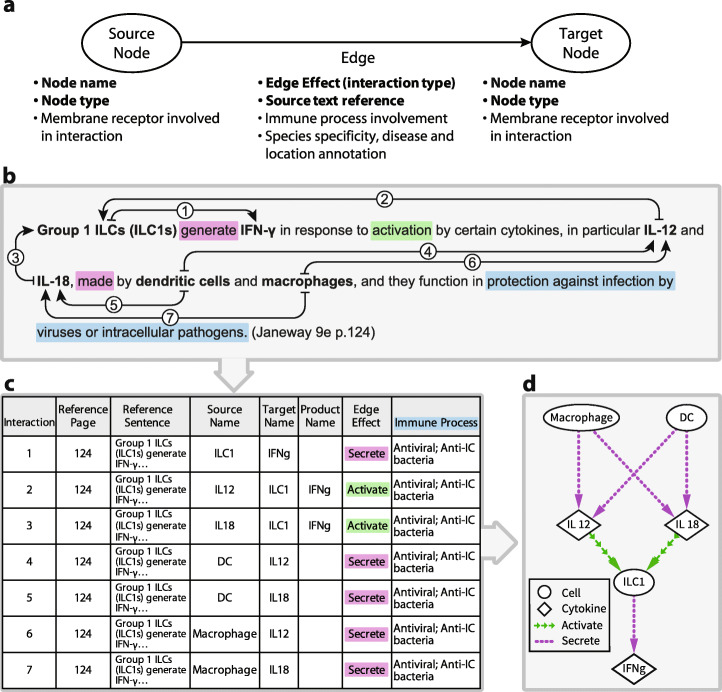
ImmunoGlobe is a directional immune interaction network that was constructed by manually codifying immune interactions described in the Janeway’s Immunobiology 9e textbook. **a** Schematic showing information recorded for each interaction. Each interaction is composed of at least a source node, target node, edge effect and source text reference. Bold text indicates required information for each edge; other points were recorded when available. **b** An example sentence showing the codification process. Seven interactions described in this sentence are annotated, with arrows originating at each source node and ending at each target node. Numbers on the arrows correspond to the “Interaction” column in 1c. Highlight colors of words in 1b correspond to the highlight colors in 1c. **c** The information extracted from sentence 1b is recorded into a network table. Each interaction between two nodes is recorded in its own row. Some rows have more detail than others, but all contain the required information (detailed in 1a). **d** The network table is used to generate a graphical representation of the described immune interactions. The entirety of the Janeway textbook was codified as illustrated here

Detailed information about 253 immune system components (nodes) and the nature of each directional interaction was recorded into a network table (Table S1). Nodes are general representations of each immune component and do not represent particular samples. For each interaction (edge), we extracted the names of the source and target nodes, the direction and type of the interaction, and the source of the data in the textbook (Fig. 1a). Additional information, such as the receptors involved, the activation states of the source and target nodes, and the immune process in which a given edge participates were recorded if available. This codification of the textbook was repeated twice and verified by an independent panel of reviewers.

A table (Table S2) designating node attributes was also generated to provide functional detail about each individual node. Each node was categorized into one of five types reflecting its identity: cell, cytokine, antibody, effector molecule, or antigen. A subtype was further assigned to reflect the function of each node. Of the 2799 interactions extracted (Table S1), 1112 were unique (Table S3). These interactions linked 253 nodes.

An example of the type of information used for construction of the network is presented in Fig. 1b. Analysis of this sentence reveals seven individual edges (interactions) between six distinct nodes (immune system components) (Fig. 1c), which were used to generate a graphical network (Fig. 1d). Although the amount of information provided by the sentence and the graphical network is identical, the graphical network formalizes the mechanistic relationships between the nodes, and enables the application of graph theory and network analysis principles to immunology.

**Fig. 2:**
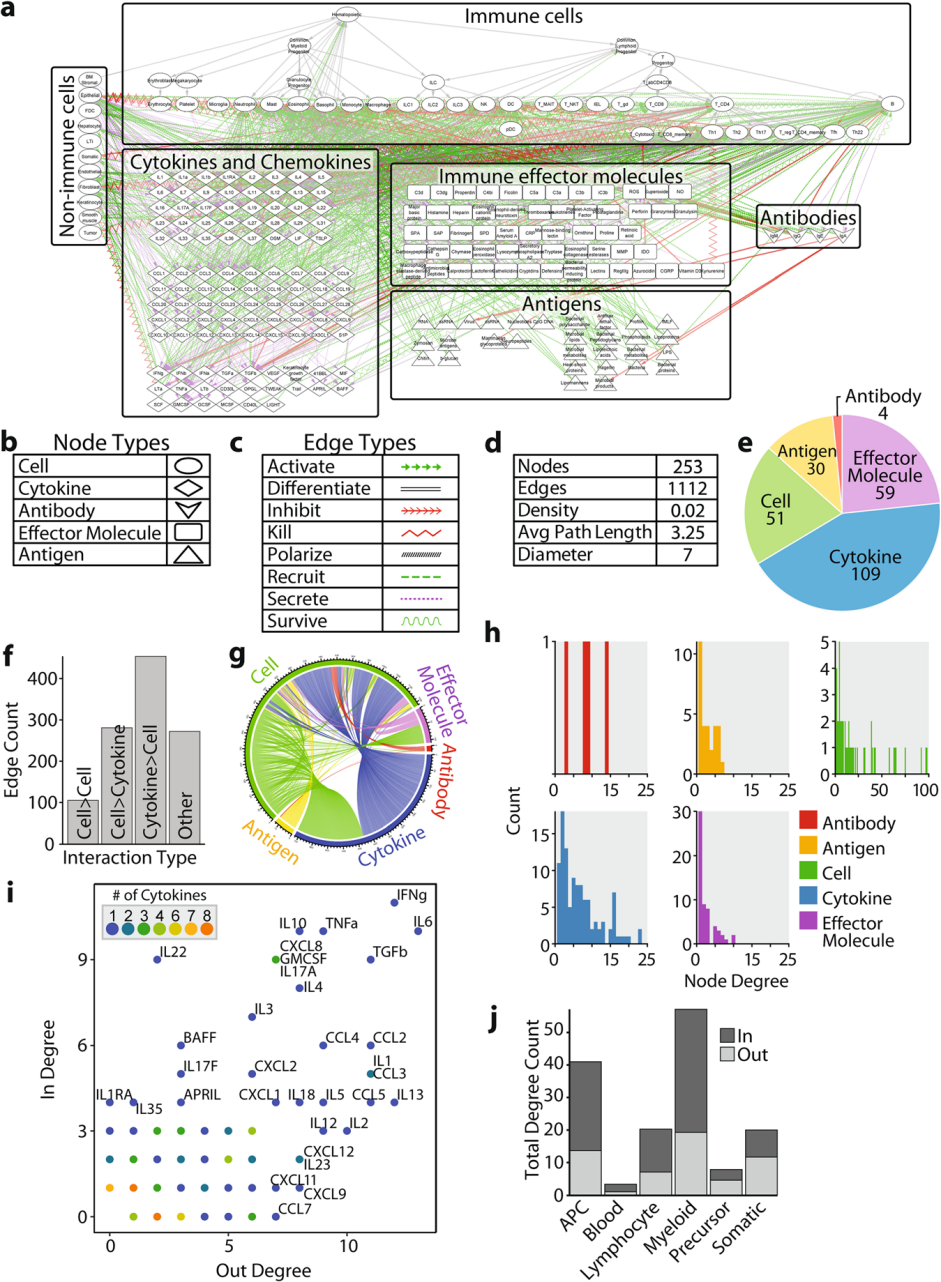
Network analysis of ImmunoGlobe recapitulates known features of the immune system. **a** A visualization of the ImmunoGlobe immune interaction network, with immune cells organized by hematopoietic lineage and other nodes grouped according to node type. Interactions between the nodes are shown as colored edges. **b** A legend showing the shapes representing each node type. **c** A legend showing the line shapes representing each edge type. **d** Summary characteristics of the immune interaction network. Number of nodes and edges are shown, and density, average path length throughout the network, and diameter of the network were calculated. **e** A pie chart showing the counts of each node type. **f** A bar graph showing the number of directional edges between different node types. The majority of interactions are between cells and cytokines. **g** Visualization of the number of edges between all node types. Each chord represents one directional interaction and is colored by node type of the source node. **h** Histograms showing the total degree distribution of each node type in the network. Each count on the Y axis represents one node. For all node types, the degree distributions skew right. **i** A scatter plot showing the in and out degrees of various cytokines. Points are colored by the number of cytokines with that combination of in- and out-degree. Cytokines with higher degrees are labeled. **j** A bar plot showing the degrees of various cell types. The height of the bar represents the total degree, with in and out degrees shown by fill color

The edge list and node attributes table were used to generate ImmunoGlobe, a graphical immune interaction network model (Fig. 2a). ImmunoGlobe was manually organized to group nodes according to function, with node type indicated by shape (Fig. 2b). Immune cells are at the top, organized according to the differentiation tree from a common hematopoietic stem cell [19]. Innate immune cells are on the left, and adaptive immune cells are on the right. Non-immune cells that interact with the immune system are collected in a column on the left. Cytokines are grouped together, separated into subgroups of interleukins, chemokines, and other cytokines. Immune effector molecules are grouped together and further clustered by subtype (e.g. Complement, reactive oxygen species). Antigens (foreign or pathogenic molecules that can stimulate an immune response) are shown at the bottom of the network. Antibody isotypes are shown on the right. Different edge types are represented by lines of different colors and styles, detailed in Fig. 2c. Edge types that are considered positive interactions (i.e., activate, recruit, or promote survival) are in green. Negative interactions (i.e., inhibit, kill) are in red. Secrete is in purple. Other edges (differentiate, polarize) are in grey. Definitions of the edge types can be found in Note S2. ImmunoGlobe thus provides a visual catalog of directional interactions between immune components and is available as an interactive network for download (File S1) and online at www.immunoglobe.org.

### The immune network model recapitulates known features of the immune system

A high-level analysis of the ImmunoGlobe network confirms known features of the human immune system, providing confidence that the topology and characteristics of this network accurately reflect our prior knowledge of immune system functioning. Most of the nodes in the network are cytokines (*n* = 109), followed by cells (*n* = 51), effector molecules (*n* = 59), antigens of various types (*n* = 30), and antibodies (*n* = 4) (Fig. 2e). The immune interaction network is large with 253 components (nodes) and 1112 interactions (edges) but has a low density of 0.02, meaning that only 2% of all possible edges in the network actually exist (Fig. 2d). This low density reflects specificity in the action of immune components, as a single node with excessively high connectivity could lead to pathologic immune responses if it were to become dysfunctional [20]. The network diameter of 7 indicates that the longest path between any two nodes is 7 steps. The average path length of the network is 3.25: It takes on average 3.25 steps along existing directional edges (interactions) to connect any two randomly selected nodes. This is shorter than would be expected by a random graph (Fig. S3), indicating that the network structure allows the rapid dissemination of information across its components [21], which is critical in the timely initiation of immune responses [22] (Fig. 2d).

The most common edges in the immune network describe the effects of cytokines on cells. The second most frequent edge type is cells secreting cytokines, followed by direct cell to cell interactions. The final category captures all edges involving antibodies, effector molecules, and antigens (Fig. 2f). The “Other” category in Fig. 2f groups together interactions between immune cells and effector molecules, antigens, and antibodies. A visualization of the interactions between all node types shows that cells are involved in over half of the total edges (Fig. 2g).

The degree counts, which measure the number of edges a node has, recapitulate prior knowledge as well. The degree distribution of the immune network skews right (Fig. 2h), showing that most nodes have relatively low degree, although there are a number of highly connected nodes. We examined the degrees of cytokine nodes by plotting the number of connections in versus the number of connections out for each individual cytokine (Fig. 2i). The number of connections in, or the “in” degree, reflects how many cell types secrete that cytokine, and “out” degree reflect the nodes that the cytokine influences. Some cytokines have low degrees and thus are highly specific: These cytokines are either secreted by or affect few cell types, whereas others with high degrees are secreted by or act upon many types of cells. The cytokines with the highest degrees are those related to inflammation (e.g. IFNγ, TNFα) and immunosuppression (e.g. TGFβ, IL10), which are relatively nonspecific processes that require broad activity across multiple modules of the immune system [23]. These processes are both initiated by many cell types and affect many immune cell types.

We next examined the degrees of the cell nodes (Fig. 2j). Cells have the highest degree of all node types because their functions are versatile, and cells can have different (and sometimes even opposing) responses depending on their physiologic context [24]. Cells carry out these varying functions by interfacing with and producing different components of the immune system. Antigen-presenting cells (APCs; here referring to dendritic cells, as described in Note S1) both sense a wide range of inputs and express or secrete numerous immune cell effectors [25]. Myeloid cells (including granulocytes), whose primary responsibility is to sense and respond rapidly to threats from the environment, have high “in” degrees but lower “out” degrees, reflecting their limited effector mechanisms [26]. Lymphocytes, the main effectors of the adaptive immune system, have lower degrees than other immune cells, reflecting their specialized and antigen-specific functions [27]. Immune cell precursors have low “in” degrees and slightly higher “out” degrees, reflecting their sensing of specialized growth and differentiation signals and their subsequent differentiation into mature immune cell subsets [19].

### ImmunoGlobe accurately represents multi-step immunologic mechanisms

**Fig. 3:**
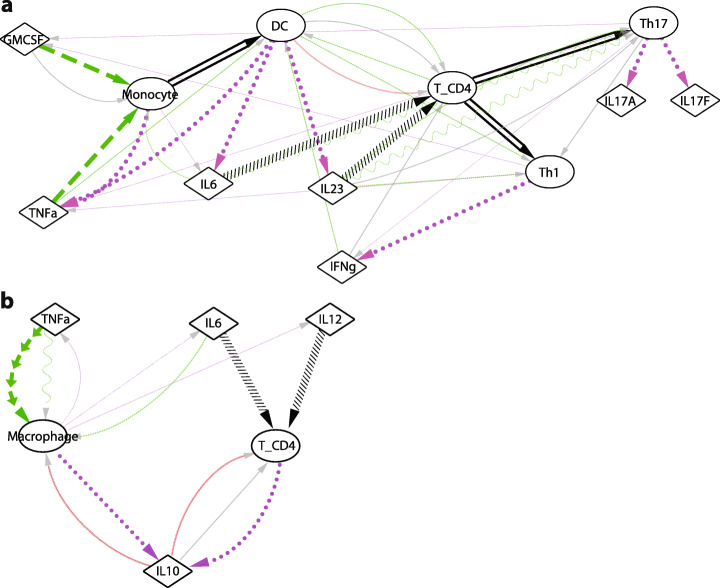
The ImmunoGlobe network model accurately reflects multi-step immunologic mechanisms. **a** Visualization of the pathway described in Iwamoto et al. Bold edges are those described in the paper, while transparent edges are additional interactions between the involved nodes that are found in ImmunoGlobe. **b** Visualization of the pathway described in Daftarian et al. Again, bold edges are those described in the paper, while transparent edges are additional interactions between the involved nodes that are found in ImmunoGlobe

One potential value of the ImmunoGlobe network lies in its capacity to uncover novel multi-step immune pathways. To test this, we performed two case studies of multi-step pathways assembled from individual network interactions to determine if there was evidence for them in the literature. Iwamoto et al. [28] reported that activation of monocyte-derived dendritic cells by TNFα and GMCSF influences their capacity to induce differentiation of CD4^+^ T cells into Th1 and Th17 cells (Fig. 3a). Although this particular pathway was not described in the textbook it exists in the network because the eleven cell types and cytokines involved exist as nodes in ImmunoGlobe, and 13 of the 14 interactions comprising it were described in other contexts in the textbook. Only one of the 14 interactions reported by these authors was absent in ImmunoGlobe (secretion of IL23 by monocytes). ImmunoGlobe also identifies several additional interactions between these nodes not reported in the Iwamoto paper. In the second study, Daftarian et al. [29] reported that IL10 secretion is enhanced in CD4^+^ T cells by the cytokines IL6 and IL12, and in monocytes by TNFα (Fig. 3b). In the ImmunoGlobe network, all edges described in the paper are present, along with additional interactions between the nodes not described in the paper. The abstracts for both papers are included in Note S3. Thus, ImmunoGlobe links interactions reported individually in the textbook into more extensive pathways supported by experimental evidence but not explicitly described in the source text. This illustrates the comprehensiveness of the network despite its being based on a single source text, and suggests that the network can be mined for previously unknown or unaccounted for interactions and pathways of interest.

### Mouse and human immune systems differ largely in the properties of their respective immune system components

**Fig. 4:**
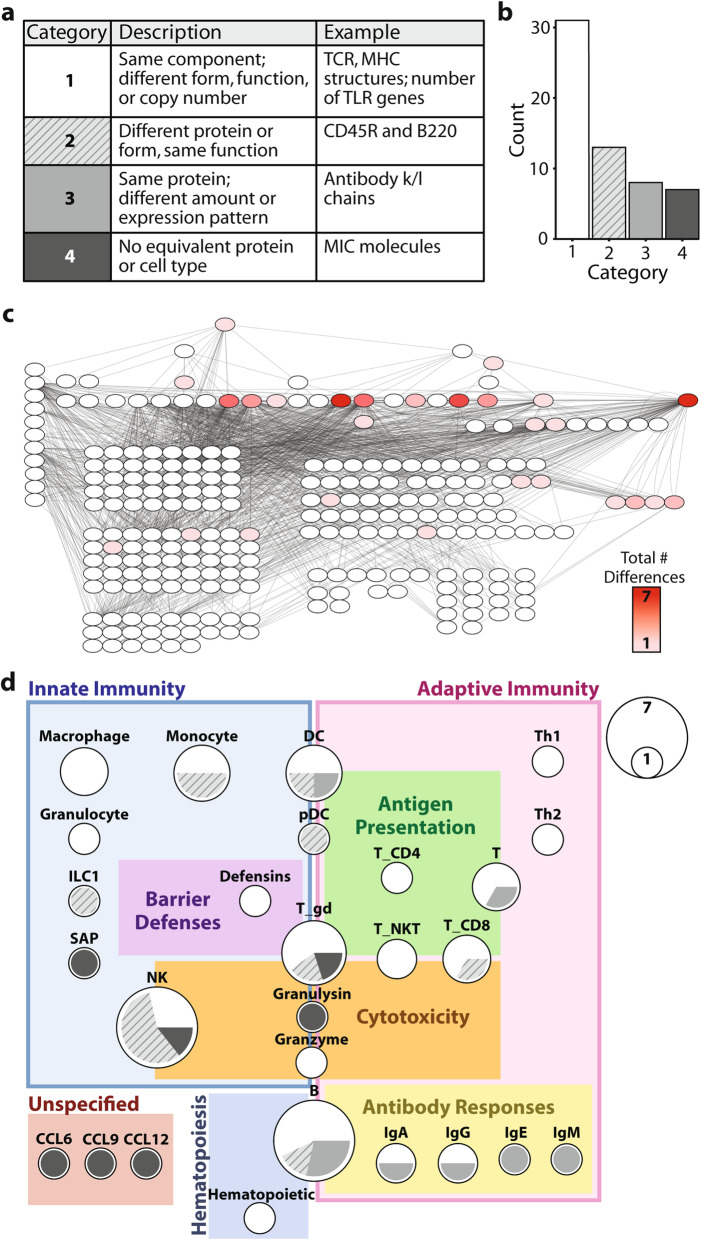
Examination of species-specific differences in mouse and human immune systems. **a** Each difference between mouse and human immune components described in Janeway was recorded and classified into one of four categories. The coloring of each category is consistent across 4a, 4b, and 4d. **b** Bar graph showing the frequency of each difference category. **c** A network visualization of ImmunoGlobe highlighting the concentration of species-specific differences in immune cells. Intensity of node color reflects the total number of differences affecting that node’s function in the immune system. **d** A visualization of the immune processes and specific nodes that differ between mouse and human immune systems. The boxes represent immune processes and are sized according to the number of species-specific immune differences affecting that process. Nodes are sized to reflect the number of differences affecting each node, and are positioned according to the process in which its differences are involved. The coloring of each node shows which proportion of differences affecting that node belong to each of the four categories described in (**a**)

Next we used ImmunoGlobe to investigate whether differences between mouse and human immune systems are reflected in the immune network structure. Each mention of a difference between mouse and human immune components (including cells, proteins, or molecules) described in *Janeway’s Immunobiology* was classified into one of four categories (Table S4) and annotated with the nodes and immune processes affected. We classified differences in node properties into four categories (Fig. 4a). Category 1 differences are those in which the component is the same between mouse and human, but form, function, or copy number differs. Category 2 are different components that perform equivalent functions. Category 3 differences are those in which the components are identical, but their levels or expression patterns differ. Category 4 are components that have no equivalent in one of the species. The most common differences between mouse and human immune components were those in Category 1 (Fig. 4b), with Category 4 being the least common. This predominance of subtle differences between the species highlights the common origin of their immune systems [30]. Indeed, the Category 4 differences (CCL6, CCL9, CCL12, SAP, and dendritic epidermal T cells are found only in mice, Granulysin and MIC molecules are found only in humans) all affect innate immune functions such as inflammation and barrier immunity, likely reflecting the different evolutionary pressures encountered by each species since their divergence [31].

Figure 4c shows the distribution of species-specific differences across the immune network, with the specific nodes and immune processes affected detailed in Fig. 4d. The differences between human and mouse affect both the innate and adaptive arms of the immune system, as well as some effector molecules (defensins, granulysin, acute phase molecule SAP) and chemokines (CCL12, CCL8, and CCL9). There are several differences in components involved in antigen presentation, including in the sequences and structures of MHC/HLA molecules, T cell receptors, the structures of antibodies, and the ratios of antibody isotypes. The ratios of circulating immune cells as well as the specific surface markers of various immune cell types differ as well. Innate immune recognition differs in the Toll-like receptors, antimicrobial molecules and enzymes that exist in each species, as well as activation control of B and NK cells. The nodes with the largest number of species-specific differences are those that represent B cells and NK cells. For B cells, these differences include differences in the positioning and sequences of the genes encoding HLA molecules, the structures of the HLA molecules, the effect of cytokines such as IL7 and TSLP on developing B cells, the surface markers that differentiate B cells, the process of recombination of the B cell receptor, and the expression of Toll-like receptors on naïve B cells. For NK cells, the differences impact their role in innate immunity, particularly in antigen recognition and cytotoxicity.

We expected that there would be differences in network structures between mice and humans based on the difficulty in translating immunomodulatory therapies between the species, but instead found that the 59 differences related instead to properties of the nodes themselves, largely in what activates the different immune components and how they are activated. The edges between the nodes do not appear to differ. For example, while TLR expression can be found in B cells of both species, they are expressed in naïve B cells constitutively in mice but only after BCR stimulation in humans [32], and the MIC and KIR genes involved in NK activation in humans are not found in mice [18]. These changes affect the reactivity of immune components rather than their interactions with other parts of the immune system.

### Immune network structure can be used to examine the network effects of immune stimuli

To demonstrate the potential application of ImmunoGlobe in helping to interpret experimental data, we performed a mass cytometry experiment to see whether we could use the immune network structure to identify a relationship between network characteristics and the strength of immune cell activation in response to stimuli. Briefly, spleens were harvested from 4 wild-type B6 mice, and whole splenocytes were incubated with LPS, TNFα, or IFNγ for 8 h, after which they were stained with a panel of antibodies that recognize phenotypic markers of major immune cell types as well as several markers known to shift in expression with activation (Fig. S1). We calculated a composite activation score for each combination of cell type and stimulus by finding the difference in average expression of each activation marker between stimulated and unstimulated, then summing across all activation markers for each cell type.

**Fig. 5:**
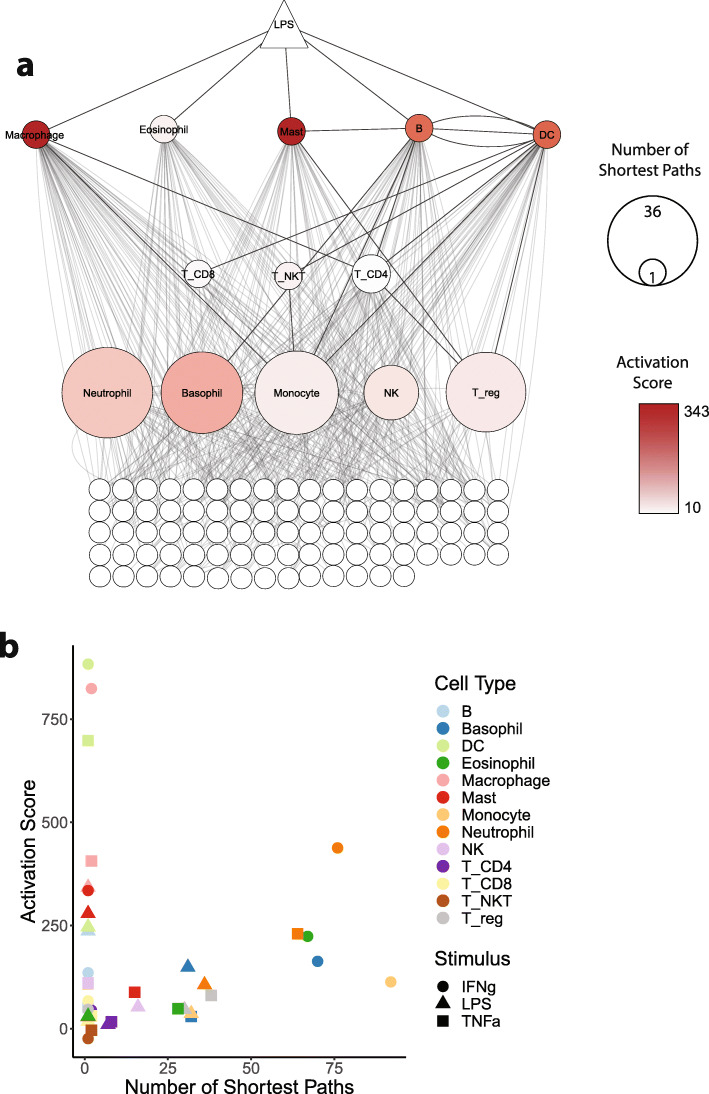
Immune network structure can be used to examine the network effects of immune stimuli. **a** A network visualization of the nodes involved in the immune response to LPS. Immune cells involved are arranged in layers corresponding to their degree of connection to the stimulus, with other interacting immune components grouped together at the bottom. Direct cell:cell edges are shown in darker grey, with all other edges involved in response to LPS shown in light grey. Immune cell node size corresponds to the number of paths between the stimulus and cell, and node color corresponds to activation score of the cell. **b** A scatter plot showing a positive correlation between the number of shortest paths that exist between a stimulus and a cell and the activation score of that cell. Data points are colored according to immune cell type and shaped according to stimulus

We hypothesized that activation scores would be highest for cell types directly activated by a given stimulus, with a decrease as the number of intermediates between the stimulus and cell type increased. Our findings broadly support this hypothesis (Fig. 5a). One notable exception is the low activation score of T cell subsets, which is likely because no antigen-specific stimuli or costimulatory signals were included in the experimental conditions.

With the exception of cells directly activated by a given stimulus, the distance (defined as the number of steps comprising the shortest path) between stimulus and cell was not correlated with activation score (Fig. S2). Rather, we found that for cells not directly activated by a stimulus, the number of shortest paths between a stimulus and cell type showed a positive correlation with that cell type’s activation score (Fig. 5b), with a Pearson’s correlation coefficient of 0.55 (*p*-value 0.007). To quantify how likely one was to observe a correlation coefficient of 0.55 or stronger at random, we performed a permutation test which gave an empirical p-value of 0.009. Eosinophils (dark green) and neutrophils (dark orange) are the best examples (Fig. 5b), with the strongest relationships between the number of shortest paths and activation score. Cell types directly activated by a stimulus did not follow this correlation as they were more strongly activated, which is expected given the direct nature of the interaction. These data therefore suggest that the strength of a cell’s response to a stimulus is dependent not just on its direct responsiveness to the stimulus, but also on the number of paths that exist between the stimulus and the cell. This finding held true for all three stimuli tested in this experiment (TNFα, LPS, and IFNγ) and demonstrate that the prediction of how strongly a given immune cell will respond to a stimulus can be informed by knowledge of its place in the immune network structure.

## Discussion

Because the immune system is complex and interconnected, it is difficult to understand how changes in one component are propagated across the entire network or how they affect the higher-level immune response as a whole. Without this understanding we are unable to predict the outcome of immune interactions or precisely modulate immune responses. This compromises our ability to manage disease as we are unable to identify the most effective drug targets, predict how drugs will alter the immune response, or determine the causes for most types of drug resistance or nonresponse. Here we describe ImmunoGlobe, a network map of the immune interactome upon which network analysis tools to accomplish these goals can be built.

To provide meaningful results network analysis tools must be based upon a network map that is highly accurate, in order to correctly represent the underlying biology, and detailed, in order to provide interpretable insights into immune mechanisms. For these reasons we decided to build ImmunoGlobe by manually curating a widely-used immunology textbook. Manual curation allowed ImmunoGlobe to achieve an accuracy rate of 99.7%, compared to one analysis of nearly 100,000 text-mined interactions with an approximate accuracy of 65% [33]. Manual curation also allows for the capture of a high level of detail: each interaction in ImmunoGlobe is annotated with one of 8 interaction types, all nodes are classified by type and subtype, and context-specific information such as anatomical location, association with disease or immune process, and species-specificity was recorded whenever available. This information would be difficult to capture accurately through text-mining because the sentences and paragraphs describing it are not consistently structured. However, ImmunoGlobe also offers an opportunity here in that the text source is included alongside each edge in the network table, providing a database that could be used to train a natural language processing algorithm. Finally, information in textbooks is (to the best of our collective scientific knowledge) that which we consider to be true – it has been satisfactorily reproduced by the scientific community and reflects true physiology rather than experimental artifacts. Manually curating a reputable textbook as a source thus ensures that all interactions comprising the network are the closest thing we can get to ground truth in biology.

As a directed graph, ImmunoGlobe enables inquiries that would be difficult or impossible to achieve by searching unstructured text. For example, searching for paired source and target nodes with differing edge types identifies all instances in which a single pair of nodes has multiple types of interactions with one another (Table S6). Most of these are unsurprising; for example, it is well known that dendritic cells can activate (via MHC:TCR interactions and costimulatory molecules), polarize (by secretion of specific cytokines) [34], or inhibit (through checkpoint molecules) naïve CD4^+^ T cells [35]. However, this analysis also revealed that IgG1 can either activate [36] or inhibit [37] granulocytes depending on which cell surface receptor it binds to. Such patterns and interactions are difficult to find in unstructured text but can be quickly identified in the graph structure.

ImmunoGlobe’s graphical structure also allows the application of more complex graph theory methods from mathematics, physics, and computer science to immunology. These methods, which leverage a network’s structure to reveal properties of its component nodes and pathways, may reveal previously unknown characteristics of immune system components – for example, the identification of critical regulatory nodes (termed hubs in network science) that may represent important control points for immune pathways and mechanisms. Additional graph-based analyses, such as process enrichment and pathway tracing, can be used to identify the cells, molecules, and processes driving a given immune response. In addition, restructuring ImmunoGlobe into a directed acyclic graph will enable dynamical modeling of immune responses and statistical network analyses such as Bayesian modeling. Additional details captured in ImmunoGlobe describe other regulatory aspects of immune function, such as anatomical location, surface receptors involved, and combinatorial signaling outcomes. Computational methods leveraging these detailed network features can be used to study how immune cells integrate a variety of (often conflicting) inputs on an intracellular level to decide their overall cellular state, and to determine how a change in the function, state, or responsiveness of one immune system component propagates across the entire immune network.

Towards the goal of predicting the outcome of immune interactions, we showed that it is not just a cell’s direct responsiveness to a stimulus that determines the strength of its response, but by how many paths through the network the stimulus can activate the cell (Fig. 5b). This demonstrates the value of the immune network graph in interpreting experimental data by showing that we are better able to predict how an immune cell will respond to stimulus with prior knowledge of its place in the immune network structure. This has applications in drug discovery and therapeutic selection in that it may be possible to predict which cells or nodes are likely to respond most strongly to a given drug or drug candidate by mapping out the connections between the molecule and cell in the immune network. It also provides a new framework with which to analyze data: given data on the response of immune cells to a given drug, one can estimate the number of paths we expect to see between the two. This may become a useful tool for hypothesis generation and suggest new directions of research to complete our understanding of the immune interactome.

In mapping the differences between human and mouse immunity onto the immune network, we had hoped to identify patterns that could inform the translation of therapeutics to humans. However, we found that most differences between mice and human immune components are subtle as even though components are not identical, they perform similar functions. Human and mouse immune responses differ largely in what activates the different immune components and how they are activated (Fig. 4); the edges *between* the nodes do not appear to differ. To extend the example of TLR differences between mice and men identified by ImmunoGlobe, additional research has shown that not only are TLR expression patterns different between the species [32], but some molecules including TLR2 [38] and TLR4 [39] show species-specific differences in activation to certain stimuli. Thus, mouse and human immune cells are not necessarily activated in the same way by the same stimuli – this is an area that could benefit from additional validation in translational research. With knowledge of the areas and pathways of the immune network that are affected by species-specific differences, and further data that quantifies the difference in function, we may better understand how to translate preclinical therapies to humans.

The immune systems analysis resource most comparable to ImmunoGlobe is immuneXpresso. ImmuneXpresso is a database of directional interactions between immune cells and cytokines text-mined from abstracts available on PubMed [4]. This method increases the number of interactions by including recently-reported findings, but limits the detail that can be captured for each and results in a lower accuracy of extracted interactions. In examining the overlap between these resources (methodology described in Note S4), we found that slightly more than half of the interactions in ImmunoGlobe also exist in immuneXpresso, but that there are interactions unique to each resource as well (Fig. S4). These data demonstrate that databases based on textbooks and literature are complementary and only partially redundant, and illustrate the value ImmunoGlobe adds to currently available immune interaction resources.

## Conclusions

ImmunoGlobe is available as an interactive network map on our website, where a user-friendly interface makes it an accessible resource for exploring the interactions between immune components (Note S5). It’s also available as a detailed edgelist (Table S1) which can be made into a fully computable graph object in analysis programs like R or Python, and as a Cytoscape network (File S1) which users can personalize and use to visualize their own data by overlaying it on the immune network structure.

ImmunoGlobe represents an important tool enabling immunology researchers to better interpret their data and explain multi-step immune-related processes. In the future, as additional tools are added on top of the core network, we anticipate that it will become possible to use ImmunoGlobe to analyze, model and explain the dynamics of immune function and dysfunction. Understanding the immune mechanisms underlying health and disease will be a first step towards developing predictive diagnostics, tools to monitor disease activity, and more targeted therapeutics.

## Methods

### Immune network table creation

#### Edge list

To capture directional immune interactions, a human curator manually extracted (codified) all interactions described in the most recent edition of *Janeway’s Immunobiology* [18]. For each interaction we recorded the page number; the descriptive text (all relevant sentences if minimum required information spanned multiple sequential sentences), figure, or table from which it was extracted; the names of the source and target nodes; and the type of interaction (hereafter referred to as the edge effect). When available, we also recorded the receptor(s) involved, the activation states of the source and target nodes, any products of the interaction, the immune process being described, whether the interaction results in proliferation of the target node, and whether the interaction occurs primarily in a specific anatomical site. For interactions described multiple times, each instance was recorded. This process yielded 2799 interactions (Table S1); 1112 unique interactions remained after merging repeated mentions.

#### Network construction quality control

For quality control purposes the manual extraction process was repeated twice and the results were compared. Only nine differences between the extractions were identified for a low error rate of 0.3%; These differences were reconciled with an independent reviewer. In addition, a panel of readers were given a randomly selected set of text references and asked to independently extract the resultant immune interactions; their results matched those of the primary ImmunoGlobe edge table. Finally, a series of programmatic sense checks were also run to ensure that no nonsensical edges existed (for example, an interaction indicating the secretion of a cell).

#### Node attributes table

The node attributes table (Table S2) was created to classify and provide details on each node. The attributes captured, including Type and Subtype, were taken from mentions of each node throughout the textbook. The node types were Cell, Cytokine, Antibody, Antigen, and Effector Molecule and are designated using definitions from Janeway [18] as follows. Cytokines are secreted proteins that affect the behavior of cells upon binding to the appropriate receptor. Antibodies are immunoglobulins secreted by cells of the B cell lineage. Effector molecules are any non-cytokine molecule, such as lipid mediators and reactive oxygen species, which interact with immune components to influence their behavior. Antigens are molecules that can initiate an immune response, such as pathogens or pathogen-associated molecules (e.g., LPS, viral genomic material, and bacterial peptidoglycans). Subtype reflected the function of the node. Additional details on classification can be found in Note S1. Each cell node is linked to the official cell ontology catalog in order to provide an objective/accepted definition of each cell type. All protein cytokines and effector molecules also include a link to UNIPROT. Nodes specific to mouse or human are noted in the Species Specificity column.

#### Ontology

Because we generalized some features (including node names, immune process annotations, and locations) in order to standardize the level of detail across the network, we built an ontology to describe the classification system. This ontology (File S2) includes cells, cytokines, effector molecules, antigens, immune processes, anatomical locations, and diseases and can be used to link edges from the original extracted edge table (Table S1) to the final edge list (Table S3) used to generate ImmunoGlobe.

### Immune network analysis

#### Network analysis

The network was created and analyzed using the igraph package version 1.2.2 in R version 3.5.1. Briefly, the edge list consisting only of unique combinations of Source Node, Target Node, and Edge Effect (Table S3) along with the node attributes table (Table S2) were read into R as CSV files, assembled into a directed network, and analyzed using functions available in the igraph package.

#### Network visualization

The network visualizations were generated with Cytoscape [40] version 3.6.0 (File S1). The default visualization was generated by manually arranging nodes with immune cells on top according to their hematopoietic differentiation hierarchy. Non-immune cells, chemokines, cytokines, antibody isotypes, and effector molecules were clustered into groups according to their Node Types and Subtypes.

The website was generated using Cytoscape.js [41].

#### Mouse versus human network comparisons

We extracted every mention of a difference between components of mouse and human immune systems (Table S4). For each difference we catalogued the page and source sentences, node or nodes involved, and primary immune process involved. The differences were then classified into one of four categories, with justification for each classification included in Table S4.

Each mentioned difference was also assigned to the node with function affected by the difference. For example, differences in MIC proteins (which are expressed on epithelial cells and fibroblasts) were assigned to natural killer (NK) cells because activation of these cells is dependent upon recognition of the MIC proteins in humans and their orthologs, ligands similar to RAET1, in mice. All nodes in Fig. 5c map directly onto nodes in the ImmunoGlobe network with the exception of the T node, which refers to mentions of unspecified T cell subsets.

### Primary mouse splenocyte stimulations and mass cytometry

Tissue from each individual mouse was prepared simultaneously. Primary mouse splenocytes were stimulated with 40 ng/mL IFNγ, 40 ng/mL TNFα, or LPS 1 μg/mL, incubated in a humidified 37 °C 5% CO_2_ incubator for 8 h, washed, and fixed as reported previously [9]. Mass-tag cellular barcoding, antibody staining, analysis on a CyTOF 2 mass cytometer (Fluidigm), and data normalization were performed as previously described [42]. A gating strategy is given in Fig. S5. In accordance with generally accepted practices in the field, we analyzed 1-5 × 10^5 cells per animal, per tissue, and per time point.

## Supplementary information

**Additional file 1.** Supplemental Information (Note S1, Note S2, Note S3; Fig. S1, Fig. S2, Fig. S3, Fig. S4, Fig. S5).

**Additional file 2: Table S1.** Complete immune interaction table.

**Additional file 3: Table S2.** Node Attributes table.

**Additional file 4: Table S3.** Final Edge List used to generate ImmunoGlobe network.

**Additional file 5: Table S4.** Catalog of differences between mouse and human immune systems.

**Additional file 6: Table S5.** Search terms used to generate immuneXpresso comparison network

**Additional file 7: Table S6.** Multiple edge types between pairs of nodes

**Additional file 8: File S1.** Interactive ImmunoGlobe network as Cytoscape file

**Additional file 9: File S2.** ImmunoGlobe ontology XML file

## Data Availability

All data generated or analyzed during this study are included in this published article and its supplementary information files.
