# Supplementary material for: ImmunoGlobe: enabling systems immunology with a manually curated intercellular immune interaction network
Source: BMC Bioinformatics. 2020 Aug 10;21:346. doi: 10.1186/s12859-020-03702-3 (PMC7430879; doi:10.1186/s12859-020-03702-3)

**Note S1:** Node Classification

The decision of whether to make naïve and activated/effector cells separate nodes was informed by their descriptions in Janeway. Cells in which naïve and activated/effector versions are recognized as phenotypically and functionally different cell types (identified by different cell surface markers, expression of different transcription factors, and/or expression of different effector molecules) are represented by distinct nodes. Naïve CD4 and CD8 T cells are shown as nodes distinct from activated effector CD4 (e.g. Th1, Th2) and CD8 (Cytotoxic) T cells. For all other immune cell types the naïve and activated/effector cells are contained in the same node, with edges specific to either state captured in the State attribute.

One exception to this format is that all B cells (e.g. naïve B cells, plasmablasts, plasma cells, and memory B cells) are contained in a single node (“B” cells). The textbook did not differentiate between naïve and effector B cells as consistently as it did for T cells (the textbook includes a total of 209 mentions of “B cell”, and only 90 mentions of a specific subtype). Therefore, in order to avoid mischaracterization, any mention of B cell subtypes was generalized to “B” cell in the edge list that generated the network. A reader interested in a specific edge can refer to the sentence source or page number to identify the specific subtype of a B cell node.

Mentions of “antigen presenting cells” were taken to mean dendritic cells, as dendritic cells are what Janeway refers to as professional antigen presenting cells. Each mention was reviewed to ensure that this assumption made sense in that particular context.

**Note S2:** Edge Definitions

| **Edge Effect/Edge Type** | **Definition** |
| --- | --- |
| Activate | Source node induces activation of the target node |
| Differentiate | Source node differentiates into target node |
| Inhibit | Source node inhibits activity of target node |
| Kill | Source node induces death of target node |
| Polarize | Source node induces differentiation of target node towards specific differentiation pathway |
| Recruit | Source node causes recruitment of target node towards location of source node |
| Secrete | Source node secretes target node |
| Survive | Source node induces or encourages survival of target node |

**Note S3:** Abstracts from studies described in Figure 3

Iwamoto S, Iwai S, Tsujiyama K, Kurahashi C, Takeshita K, Naoe M, Masunaga A, Ogawa Y, Oguchi K, Miyazaki A. TNF-alpha drives human CD14+ monocytes to differentiate into CD70+ dendritic cells evoking Th1 and Th17 responses. J Immunol. 2007 Aug 1;179(3):1449-57. PubMed PMID: 17641010.

 Abstract:

Many mechanisms involving TNF-alpha, Th1 responses, and Th17 responses are implicated in chronic inflammatory autoimmune disease. Recently, the clinical impact of anti-TNF therapy on disease progression has resulted in re-evaluation of the central role of this cytokine and engendered novel concept of TNF-dependent immunity. However, the overall relationship of TNF-alpha to pathogenesis is unclear. Here, we demonstrate a TNF-dependent differentiation pathway of dendritic cells (DC) evoking Th1 and Th17 responses. CD14(+) monocytes cultured in the presence of TNF-alpha and GM-CSF converted to CD14(+) CD1a(low) adherent cells with little capacity to stimulate T cells. On stimulation by LPS, however, they produced high levels of TNF-alpha, matrix metalloproteinase (MMP)-9, and IL-23 and differentiated either into mature DC or activated macrophages (M phi). The mature DC (CD83(+) CD70(+) HLA-DR (high) CD14(low)) expressed high levels of mRNA for IL-6, IL-15, and IL-23, induced naive CD4 T cells to produce IFN-gamma and TNF-alpha, and stimulated resting CD4 T cells to secret IL-17. Intriguingly, TNF-alpha added to the monocyte culture medium determined the magnitude of LPS-induced maturation and the functions of the derived DC. In contrast, the M phi (CD14(high)CD70(+)CD83(-)HLA-DR(-)) produced large amounts of MMP-9 and TNF-alpha without exogenous TNF stimulation. These results suggest that the TNF priming of monocytes controls Th1 and Th17 responses induced by mature DC, but not inflammation induced by activated M phi. Therefore, additional stimulation of monocytes with TNF-alpha may facilitate TNF-dependent adaptive immunity together with GM-CSF-stimulated M phi-mediated innate immunity.

Daftarian PM, Kumar A, Kryworuchko M, Diaz-Mitoma F. IL-10 production is enhanced in human T cells by IL-12 and IL-6 and in monocytes by tumor necrosis factor-alpha. J Immunol. 1996 Jul 1;157(1):12-20. PubMed PMID: 8683105.

Abstract:

IL-10, an immunoregulatory cytokine produced by T cells and monocytes, inhibits the expression of inflammatory and hemopoietic cytokines as well as its own expression. To evaluate the regulation of IL-10 production by T cells and monocytes, we measured IL-10 levels by ELISA in supernatants of PHA-stimulated PBMC following depletion of either T cells or monocytes. IL-10 production was significantly down-regulated in both T cell- and monocyte-depleted PBMC compared with undepleted PBMC, and IL-10 production could be restored by the addition of monocyte-conditioned medium (supernatant of PHA-stimulated, T cell-depleted PBMC), suggesting that IL-10 production by T cells is regulated by a monokine(s) produced by activated monocytes. To further clarify the monokine(s) responsible for IL-10 induction, we stimulated monocyte-depleted PBMC, purified CD4+, and CD8+ T cells with PHA and measured IL-10 production by ELISA and semiquantitative reverse transcriptase-PCR following monokine(s) addition. Addition of IL-6 and IL-12 enhanced IL-10 production in monocyte-depleted PBMC in a dose-dependent and additive manner. Furthermore, anti-IL-6 and anti-IL-12 Abs neutralized the IL-10-inductive effect of monocyte-conditioned medium. Similarly, IL-12 and IL-6 induced IL-10 production by purified CD4+ and CD8+ T cells. With respect to regulation of IL-10 produced by monocytes, TNF-alpha was found to induce IL-10 production by resting as well as by LPS-stimulated purified monocytes/macrophages. Taken together, these findings suggest that IL-10 production by human T cells and monocytes is differentially regulated. IL-12 and/or IL-6 can induce the expression of IL-10 by PHA-stimulated T cells, whereas TNF-alpha induces IL-10 production by monocytes. Since IL-10 inhibits the production of IL-6, IL-12, and TNF-alpha, these results may indicate a potential mechanism of negative feedback regulation of the immune response.

**Figure S1:** Arcsinh-transformed median expression values for activation markers, faceted by cell type. Color corresponds to stimulus.


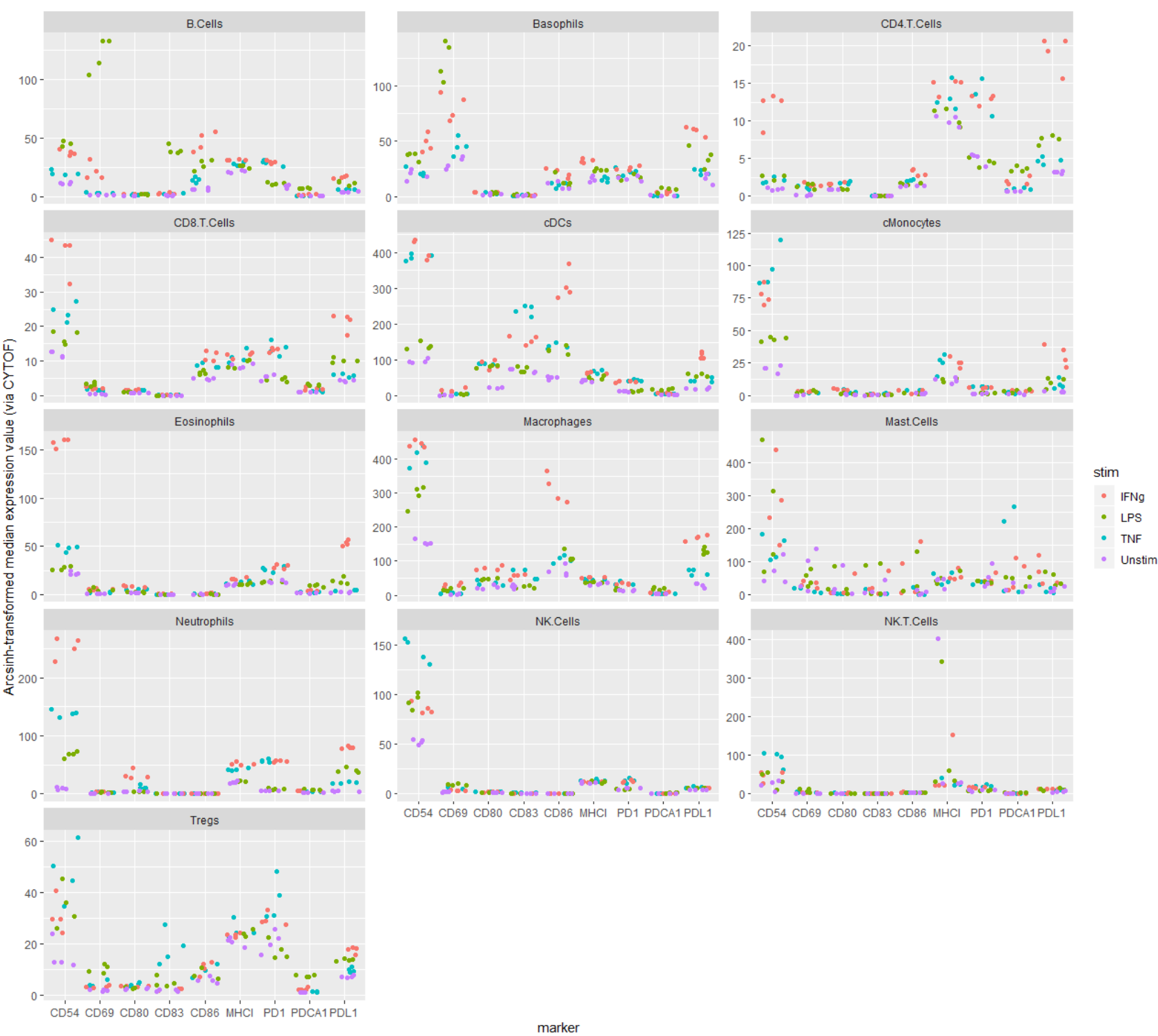


**Figure S2:** Scatterplot showing relationship between activation level and the length of shortest path between a stimulus and cell type.

**
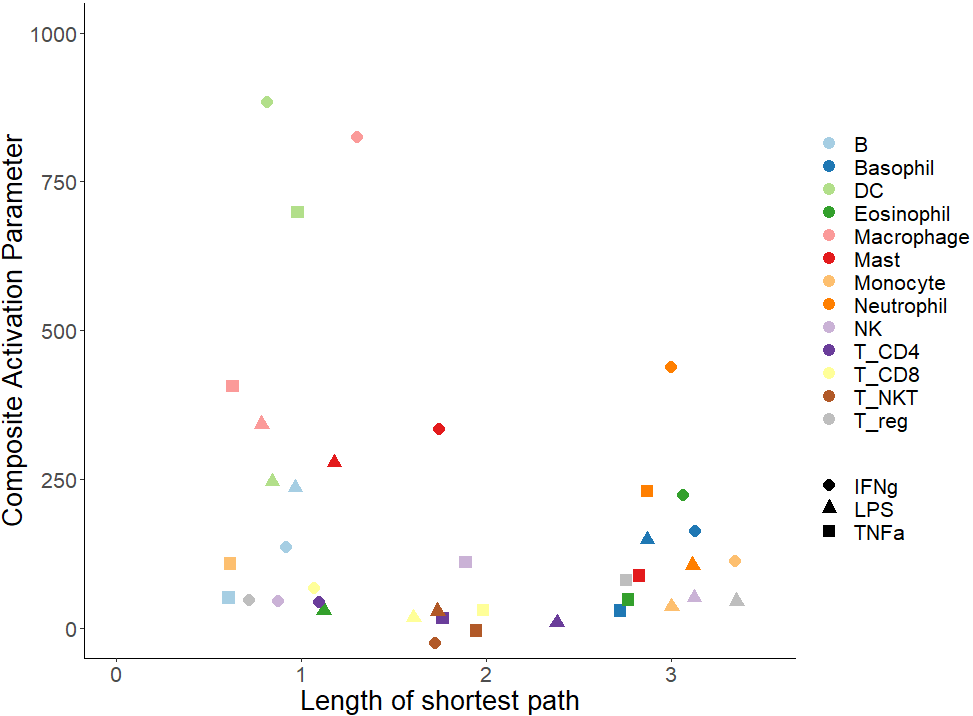
**

**Figure S3:** Histogram showing average path length of 100,000 Erdos-Renyi random graphs with the same properties (253 nodes, density of 0.02) as ImmunoGlobe. The average path length of ImmunoGlobe (3.25; indicated by a red dashed vertical line) is less than those of the random graphs.


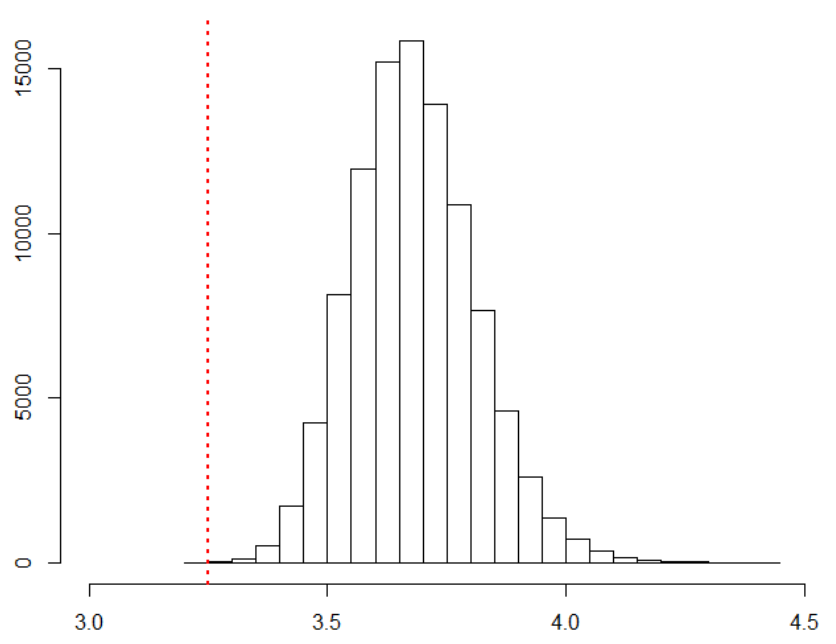


**Average path length of 100,000 random graphs**

Shortest Path Length

Frequency

p = 0.00005

**Note S4:** Comparisons of ImmunoGlobe and immuneXpresso

We downloaded all edges between cell and cytokine nodes that exist in the ImmunoGlobe network from the immuneXpresso web portal (Kveler et al., 2018). Some cell types and cytokines (for example, innate lymphoid cells) did not exist in the immuneXpresso database and therefore are not included in the networks comparing ImmunoGlobe and immuneXpresso. All cells and cytokines in ImmunoGlobe and the corresponding search term used to identify them in immuneXpresso are listed in Table S5. For purposes of this comparison only cell and cytokine nodes were included, as immuneXpresso does not contain interactions between immune cells and non-cytokine components (such as effector molecules, antigens, or antibodies).

The data downloaded from immuneXpresso for each edge included the source and target node, edge sentiment (positive, negative, or unknown), number of reference papers, and an Enrichment score. The downloaded CSV files were merged and reformatted to match the format of the ImmunoGlobe edge list.

For all visual network/graph representations, the ImmunoGlobe and immuneXpresso networks are shown with the same spatial arrangement of nodes. When edges were compared, only source node, target node, and direction of the edge was considered, as these were the only features present at the same level of detail in both networks.

**Figure S4:** Immune interactions beyond ImmunoGlobe can be examined by searching the literature via immuneXpresso.

1. A visual representation of the ImmunoGlobe network, showing immune cell and cytokine nodes arranged in concentric circles with blue edges showing interactions between the nodes. Edges unique to ImmunoGlobe are those describing cell to cell interactions, which are not included in immuneXpresso, and producers of chemokines.
2. A visual representation of interactions between ImmunoGlobe nodes as catalogued in immuneXpresso. The node set is identical to and arranged in the same layout as 6a, with interactions between the nodes shown as red edges. Edges unique to ImmuneXpresso tend to increase the connectivity of cytokines, showing both more producer and responsive immune cells for many cytokines, especially interleukins. ImmuneXpresso also contains more interactions with recently discovered cell types such as natural killer T cells, plasmacytoid dendritic cells, and plasma cells.
3. The combined ImmunoGlobe and immuneXpresso networks. Shared edges (n = 315) are shown in purple, while edges that are unique to either ImmunoGlobe (n = 292) or immuneXpresso (n = 955) shown in lighter grey.
4. Adjacency matrices of the combined networks. Each directional edge is represented by a unique point at the intersect of a source node and target node and is colored by whether the edge exists in both networks (purple) or is unique to either ImmunoGlobe (blue) or immuneXpresso (pink).

**
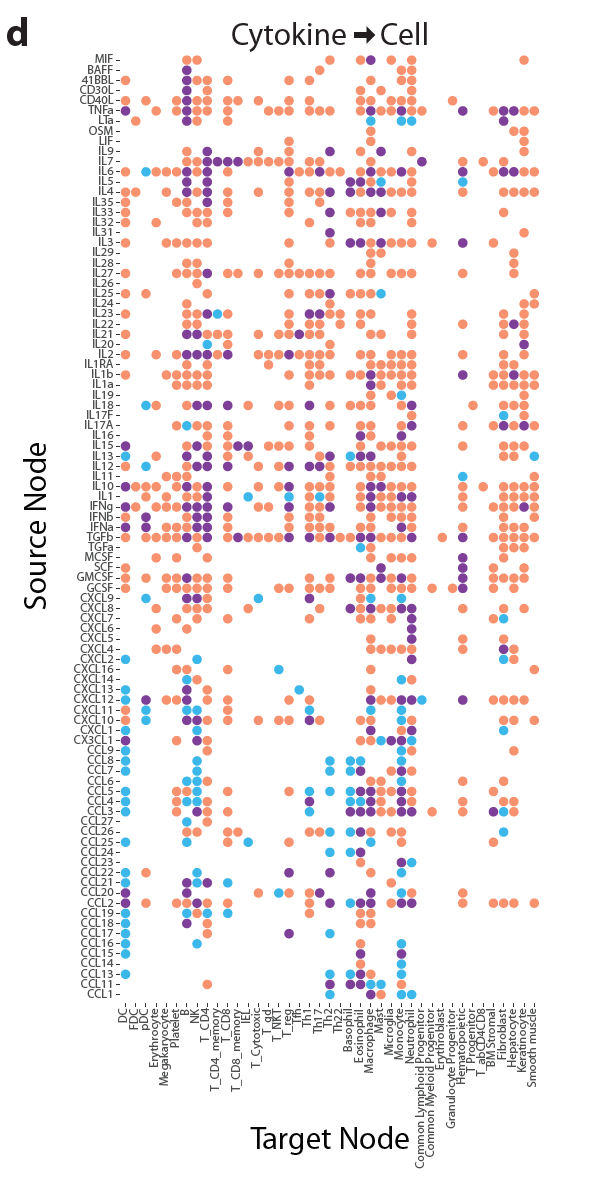
**
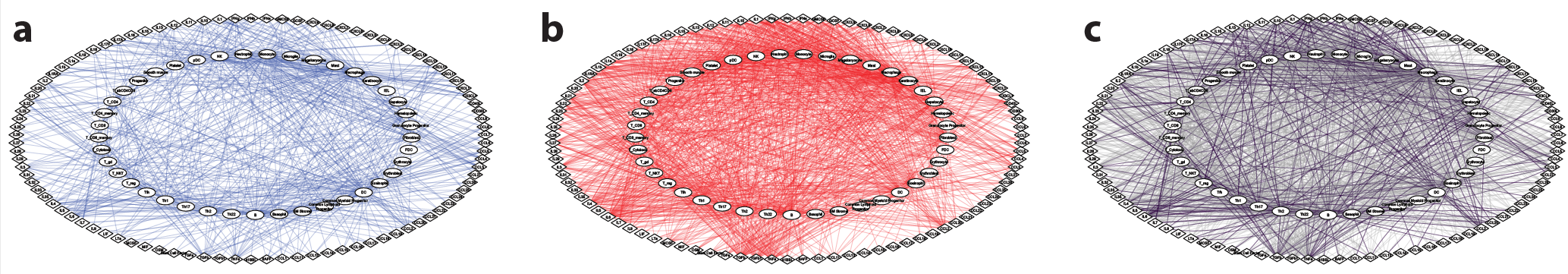


**
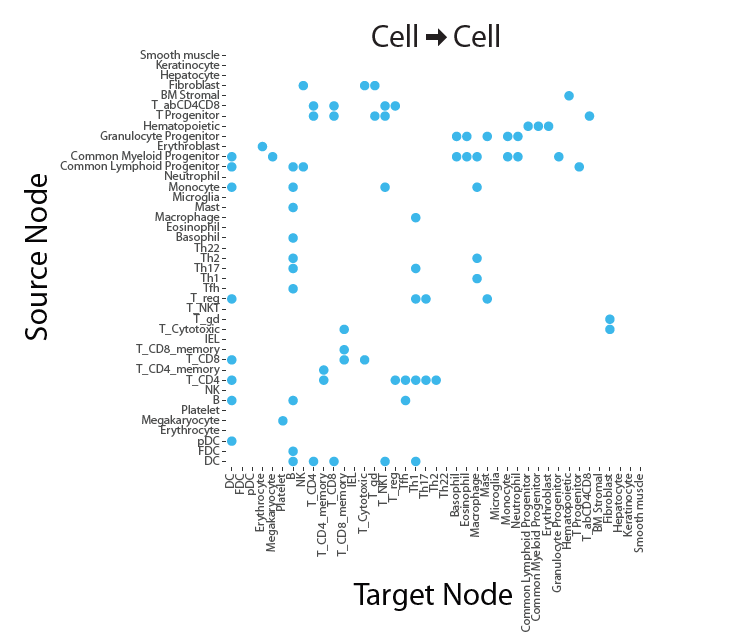
**

**
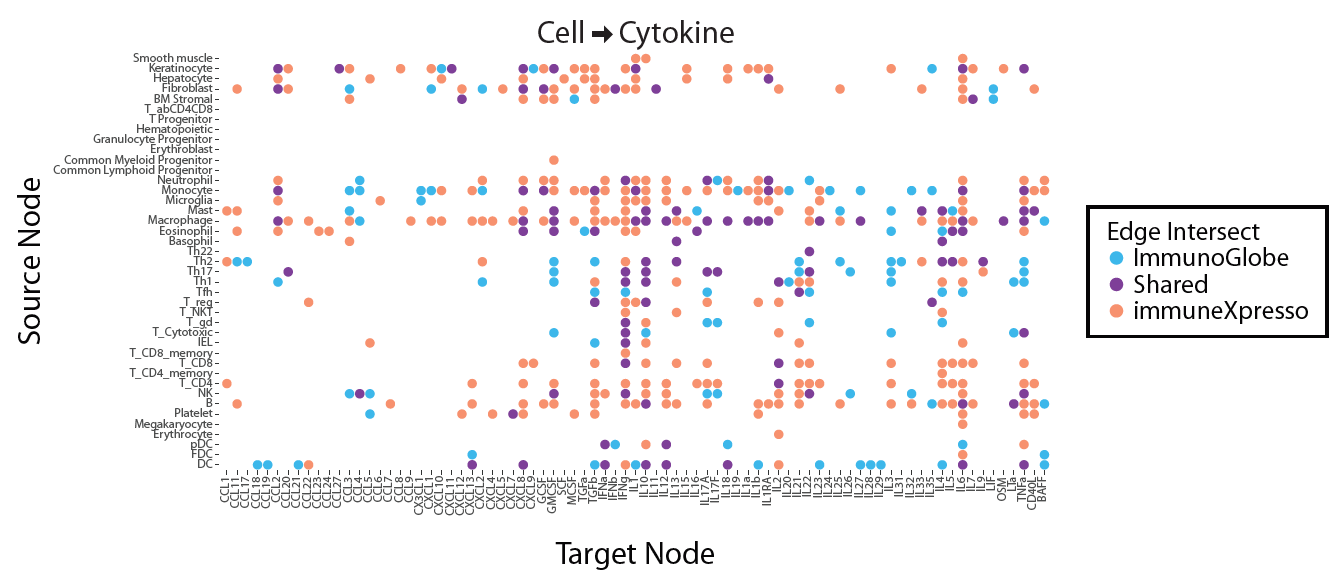
**

**Figure S5**: Gating strategy for CyTOF data.

**
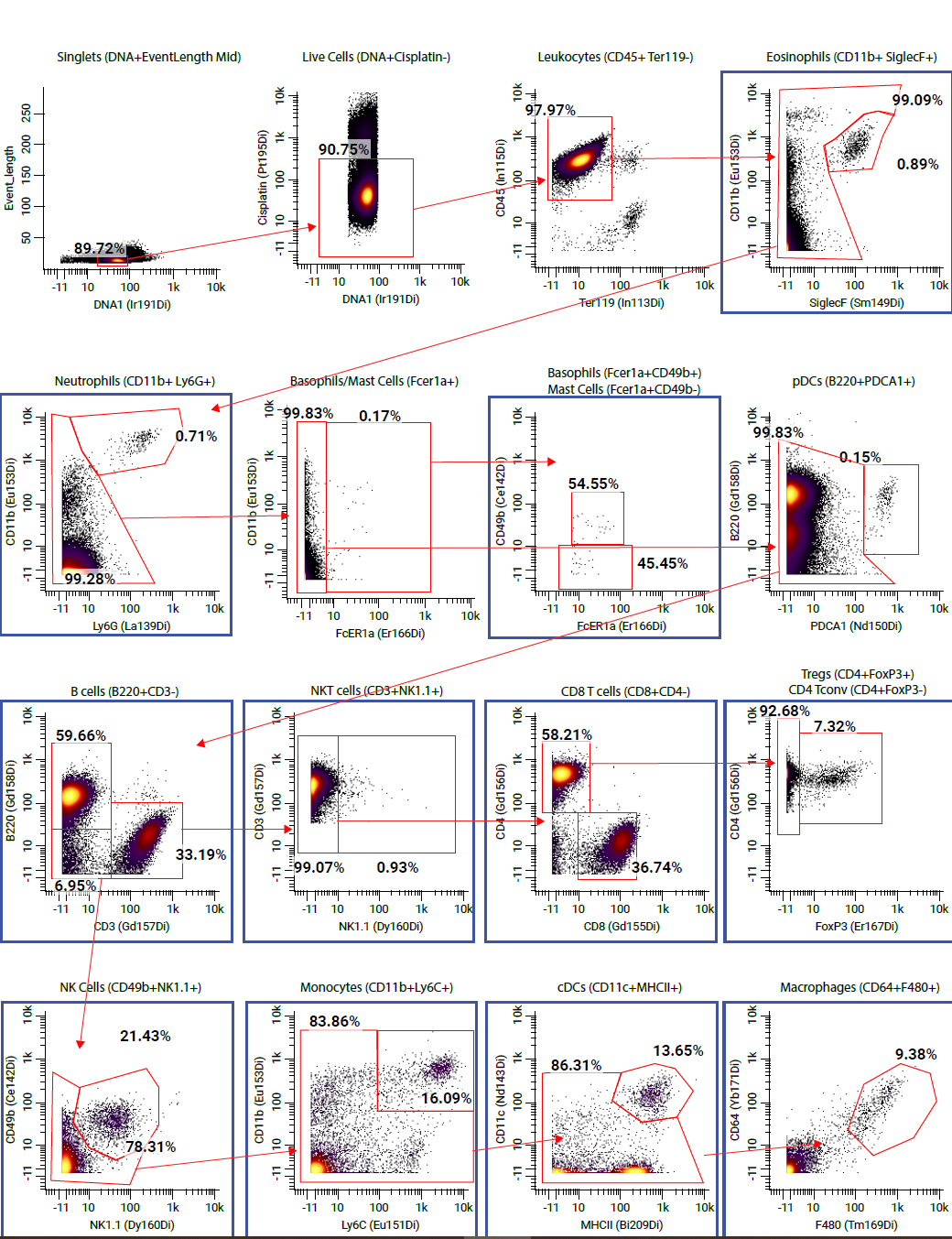
**

**Note S5:** Immunoglobe.org website tutorial

Use ImmunoGlobe’s Search, Filter, and New Network functions to find the nodes and edges describing immune interactions of interest. Below is a description of these functions, and at the bottom of the page are a few step-by-step use case examples.

**Getting Started**


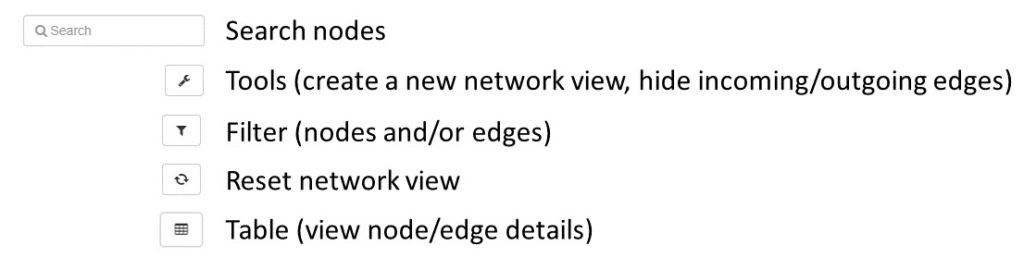


Select Nodes – Click on a node to select it (nodes will turn red when selected). Each node you click will be added to your selection. To de-select a node, simply click on it again.

Zoom with your mouse – scroll up to zoom in, scroll down to zoom out.

Pan around the network by clicking and dragging.

**Find Nodes of Interest**

Browse

The visual layout of the network is organized by grouping nodes with similar function together. You can see these groupings below:


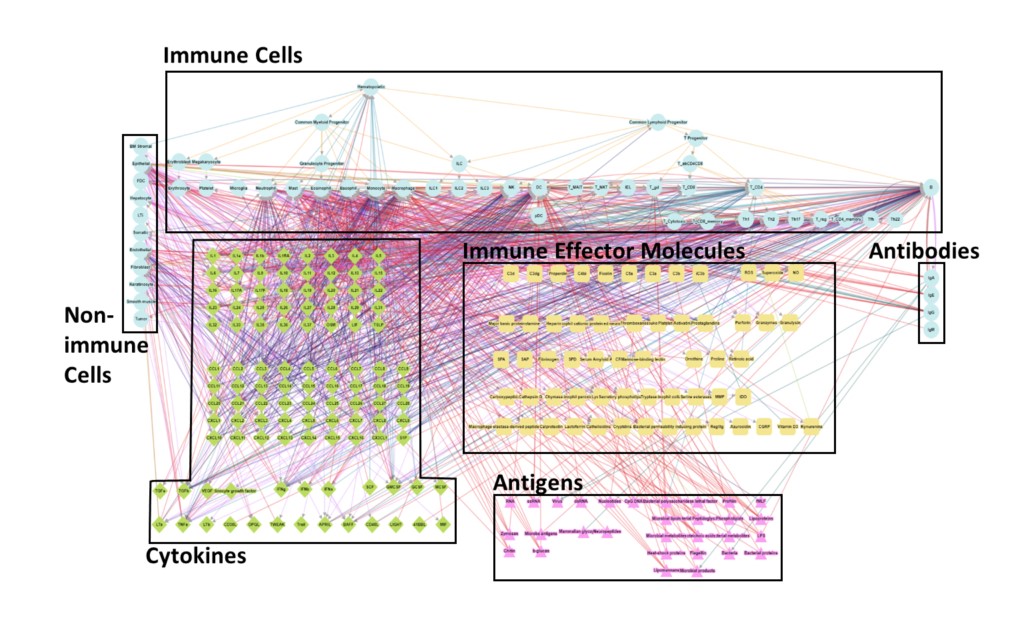


Search

To more quickly find a node of interest, use the search bar to type in the name. As you type, matching nodes will appear under the search bar, and the corresponding nodes on the network will become highlighted in red.


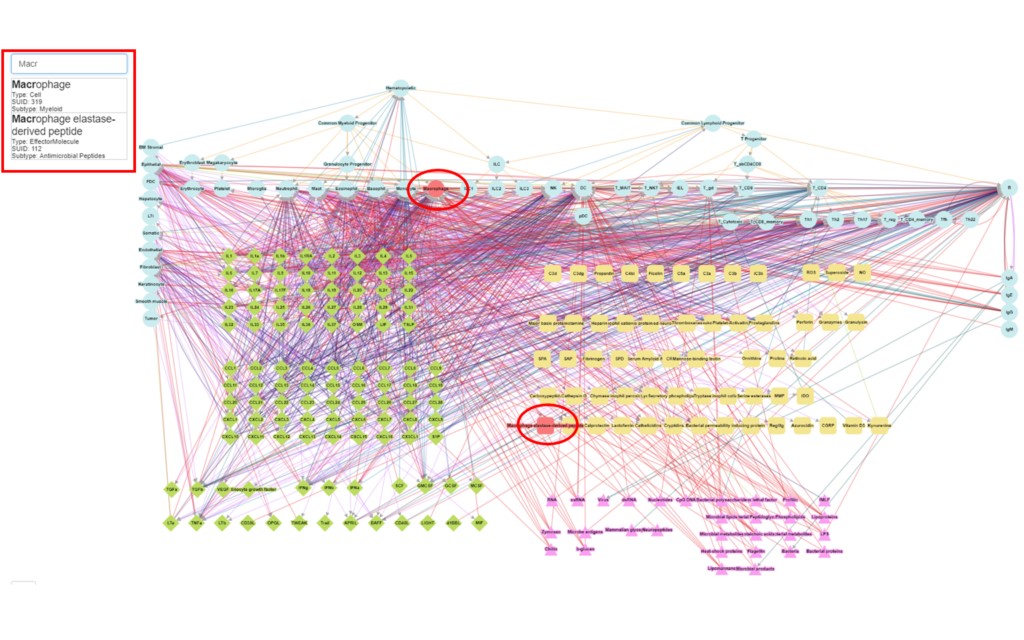


Filter

You can choose which types and subtypes of nodes to show or hide by using the filter function. Hiding a type of node will also hide all of its subtypes.

For example, if you uncheck the “cytokine” box, all cytokines will become hidden (A). If you uncheck just the “chemokine” box, the chemokines will become hidden but other cytokines (such as interleukins, growth factors, etc) will remain visible (B).
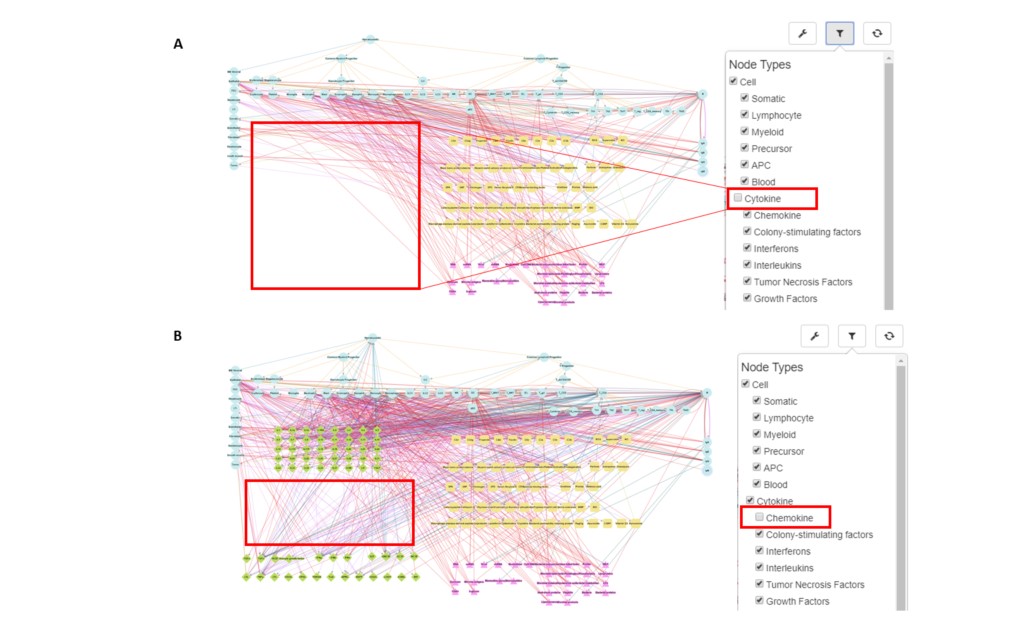


You can also filter edge types in the same menu. The Edge Type filter also serves as a legend, showing which colors represent which edge types in the network visualization.

**Create a New Network View**

Once you’ve selected your nodes of interest, you can create a new network view with only the nodes and edges you are interested in. To do this, select the node(s) you are interested in, click the Tool button, and select “New network from selected”. This will create a new network view with your selected nodes and any nodes directly connected to them.


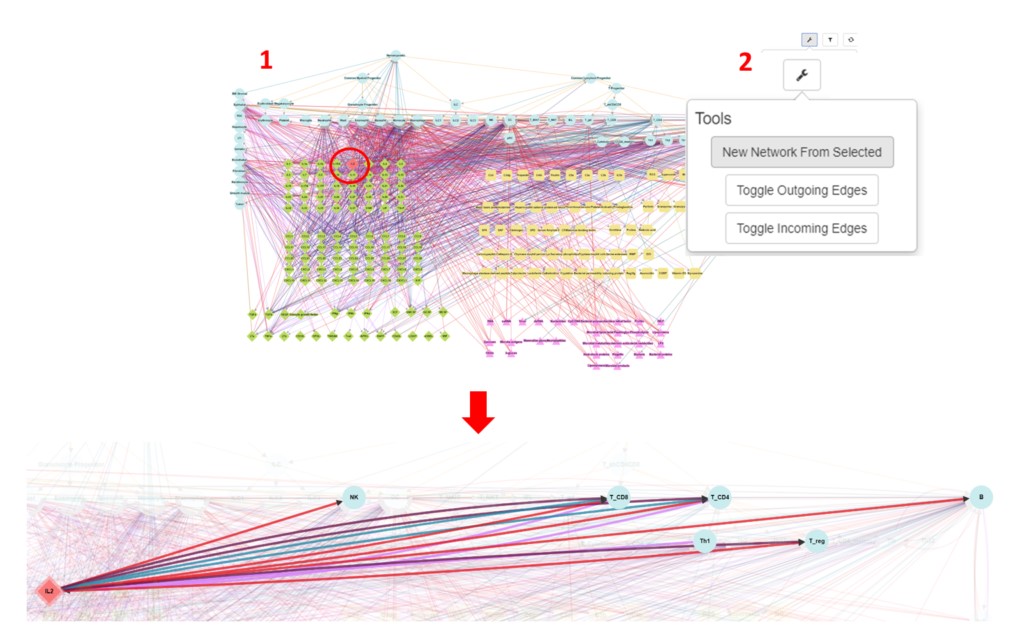


Once you have a new network view, you can toggle in/out edges to only show incoming edges (things that affect your node(s) of interest), or outgoing edges (effects your node(s) of interest have on other nodes).


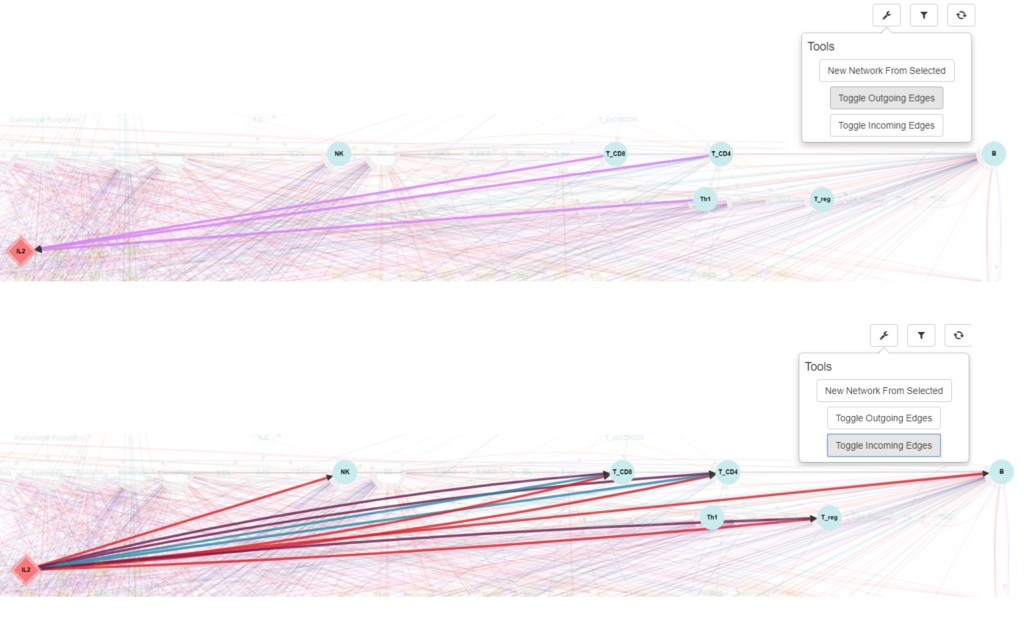


You can also use filters on your new network view to only show specific node types or edge types, leaving visible only the interactions you are interested in.

**View interaction details**

Once you’ve identified the interactions you’re interested in, you can generate a data table to view details.

Node Table

The node table shows attributes of all selected nodes (selected nodes are highlighted in red). To see details for your nodes of interest, click to select them and then click the Table button. The node table will be generated automatically and will display the node name, type, and subtype, along with a cross reference to the cell line ontology or protein database as appropriate.


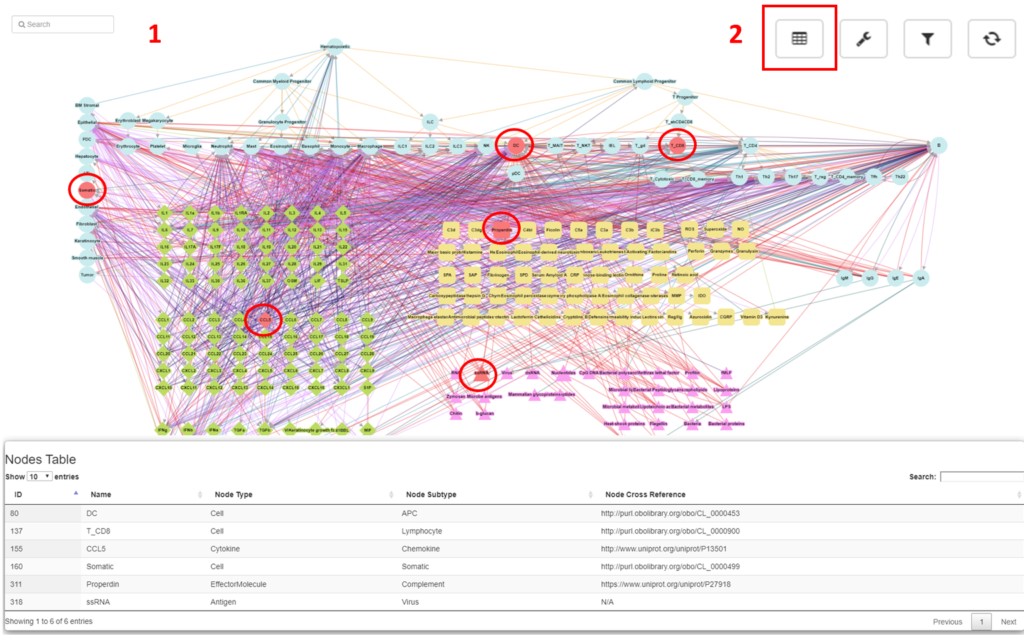


Edge Table

Because edges cannot be selected like nodes, the edge table shows attributes of all visible edges. Use the tools above to leave visible only the interactions you’re interested in. Then click the table button, and click “Load/Reload edge table” to generate the edge table.


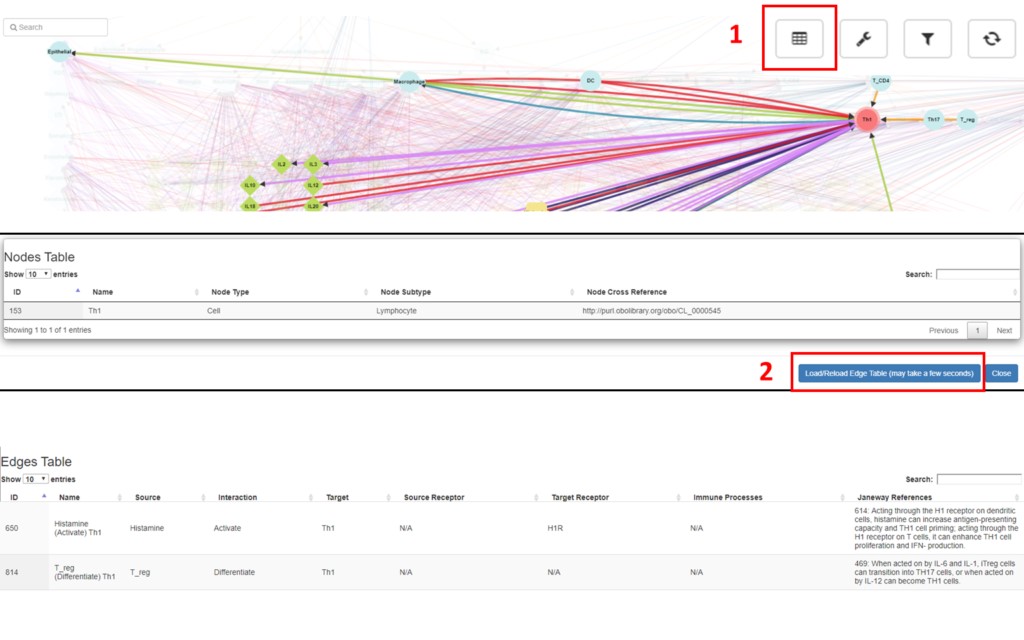


If you generate an edge table and then change the visible interactions, the edge table will not update automatically. Click “Load/Reload edge table” to re-generate an edge table that reflects what is currently on your screen.

Both Node and Edge tables can be sorted by clicking column names, and searched using the respective search bars within the tables.

**Example: Find all molecules secreted by macrophages**

1. Select the macrophage node

2. Click the “Tools” button, and click “New network from selected”

3. Click the “filter” button and unselect all edge types other than ‘Secrete’


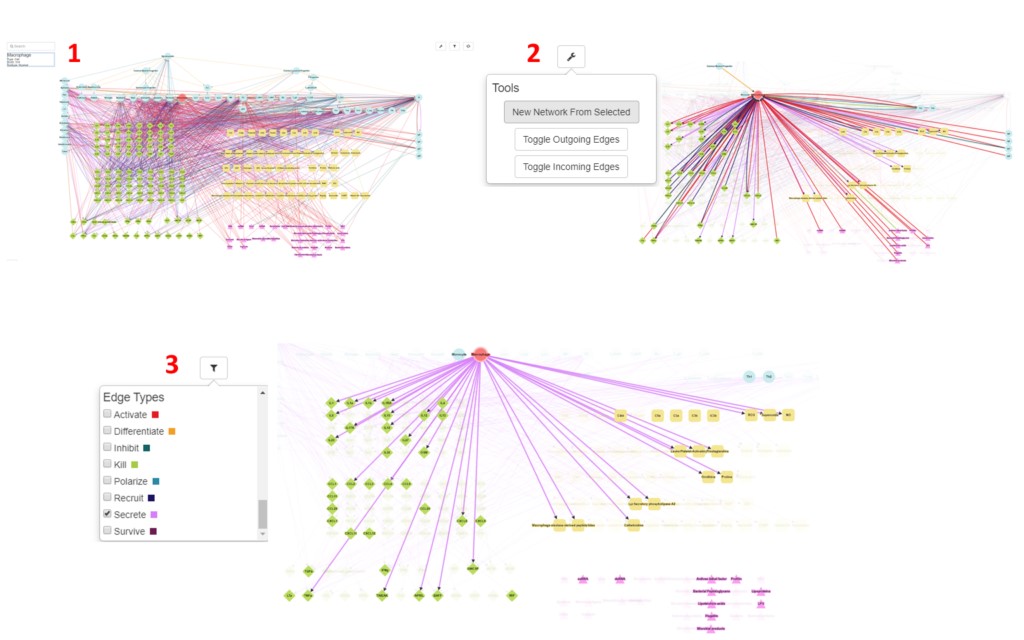


**Example: Find nodes that inhibit Dendritic Cells**

1. Select the DC node

2. Click the “tools” button and click “New network from selected”

3. Click “Toggle Outgoing Edges” to hide outgoing edges (effects of DCs on other nodes) 4. In the filter menu, uncheck all edge types other than “Inhibit”


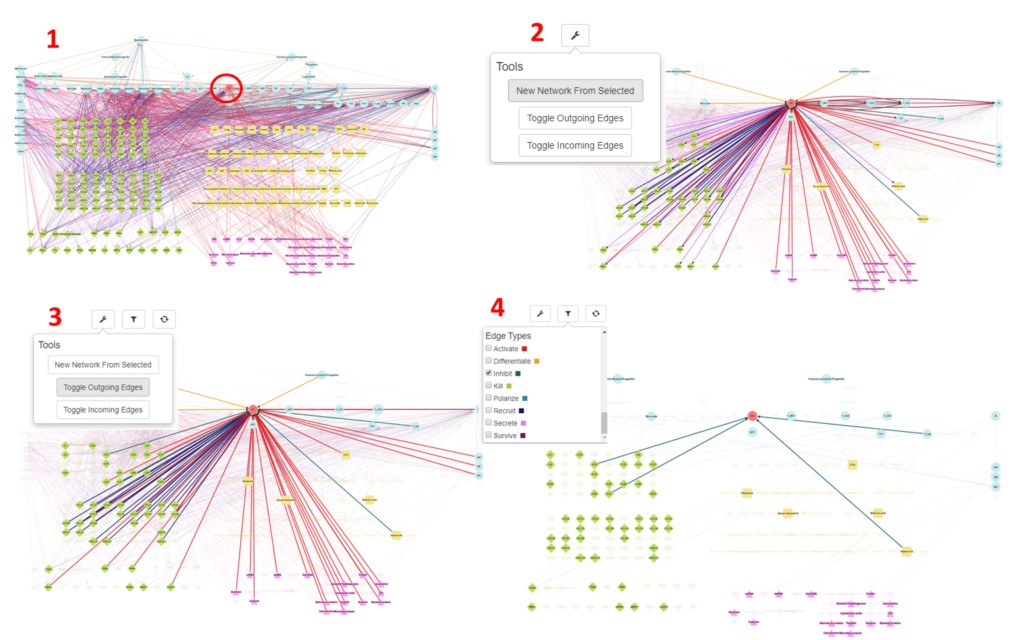

Supplement: Supplementary file 1 — Additional file 1. Supplemental Information (Note S1, Note S2, Note S3; Fig. S1, Fig. S2, Fig. S3, Fig. S4, Fig. S5). [file 12859_2020_3702_MOESM1_ESM.docx]
